# *Socrates:* A Novel N-Ethyl-N-nitrosourea-Induced Mouse Mutant with Audiogenic Epilepsy

**DOI:** 10.3390/ijms242317104

**Published:** 2023-12-04

**Authors:** Elena G. Varlamova, Ekaterina V. Borisova, Yuliya A. Evstratova, Andrew G. Newman, Vera P. Kuldaeva, Maria S. Gavrish, Elena V. Kondakova, Victor S. Tarabykin, Alexey A. Babaev, Egor A. Turovsky

**Affiliations:** 1 Institute of Cell Biophysics of the Russian Academy of Sciences, Federal Research Center “Pushchino Scientific Center for Biological Research of the Russian Academy of Sciences”, 142290 Pushchino, Russia; 1928lv@mail.ru; 2 Institute of Cell Biology and Neurobiology, Charité-Universitätsmedizin Berlin, Charitéplatz 1, 10117 Berlin, Germany; brusnichka_ne@mail.ru (E.V.B.); andrew.newman@charite.de (A.G.N.); 3 Institute of Neuroscience, Lobachevsky State University of Nizhny Novgorod, 23 Gagarin Ave., 603022 Nizhny Novgorod, Russia; verunya.rubackova@mail.ru (V.P.K.); mary_gavrish@mail.ru (M.S.G.); elen_kondakova@list.ru (E.V.K.); alexisbabaev@list.ru (A.A.B.); 4 Federal State Budgetary Educational Institution of Higher Education “MIREA—Russian Technological University”, 78, Vernadskogo Ave., 119454 Moscow, Russia; ya.evstratovayulya1@yandex.ru; 5 Research Institute of Medical Genetics, Tomsk National Research Medical Center, Russian Academy of Sciences, 10 Nab. Ushaiki, 634050 Tomsk, Russia

**Keywords:** epileptiform activity, calcium ions, signaling, receptors, gene expression, mutagenesis, neurons

## Abstract

Epilepsy is one of the common neurological diseases that affects not only adults but also infants and children. Because epilepsy has been studied for a long time, there are several pharmacologically effective anticonvulsants, which, however, are not suitable as therapy for all patients. The genesis of epilepsy has been extensively investigated in terms of its occurrence after injury and as a concomitant disease with various brain diseases, such as tumors, ischemic events, etc. However, in the last decades, there are multiple reports that both genetic and epigenetic factors play an important role in epileptogenesis. Therefore, there is a need for further identification of genes and loci that can be associated with higher susceptibility to epileptic seizures. Use of mouse knockout models of epileptogenesis is very informative, but it has its limitations. One of them is due to the fact that complete deletion of a gene is not, in many cases, similar to human epilepsy-associated syndromes. Another approach to generating mouse models of epilepsy is N-Ethyl-N-nitrosourea (ENU)-directed mutagenesis. Recently, using this approach, we generated a novel mouse strain, *soc* (*socrates*, formerly *s8-3*), with epileptiform activity. Using molecular biology methods, calcium neuroimaging, and immunocytochemistry, we were able to characterize the strain. Neurons isolated from *soc* mutant brains retain the ability to differentiate in vitro and form a network. However, *soc* mutant neurons are characterized by increased spontaneous excitation activity. They also demonstrate a high degree of Ca^2+^ activity compared to WT neurons. Additionally, they show increased expression of NMDA receptors, decreased expression of the Ca^2+^-conducting GluA2 subunit of AMPA receptors, suppressed expression of phosphoinositol 3-kinase, and BK channels of the cytoplasmic membrane involved in protection against epileptogenesis. During embryonic and postnatal development, the expression of several genes encoding ion channels is downregulated in vivo, as well. Our data indicate that *soc* mutation causes a disruption of the excitation–inhibition balance in the brain, and it can serve as a mouse model of epilepsy.

## 1. Introduction

The cerebral cortex is the main achievement of mammalian evolution [1]. The cortex forms the biological basis of human higher cognitive potential, which includes language, problem solving, reasoning, and decision making. The development of the cerebral cortex is a complex and highly organized process. Disruption of any of the overlapping steps that contribute to this process can result in a variety of developmental disorders. Many of these disorders are related to malformations of the cerebral cortex [2]. Brain malformations often cause such disorders as epilepsy, developmental delays, neurological deficits, and mental retardation in humans.

Epilepsy is one of the most socially significant brain diseases because it affects around 50 million people worldwide [3]. Based on the literature, it is reported that a percentage ranging between 5 and 50% of epilepsy patients develop the condition as a result of brain injury, tumor development, or stroke [4]. Additionally, approximately 30% of patients with epilepsy have the condition due to gene mutations. Notably, studies have demonstrated that in childhood-onset genetic epilepsies, around 80% of genetic diagnoses have potential treatment implications [5,6]. For hereditary epilepsies, there are two main pathways of their genesis—monogenic epilepsies, which occur as a result of the mutation of a single gene, and multifactorial genetic epilepsies, when multiple mutations occur and are influenced by environmental factors [7].

The main cause of epileptic seizures is believed to be a disruption in the excitation–inhibition balance. This, in turn, can be caused by various reasons, such as an impaired ratio of excitatory/inhibitory neuronal subtypes, changes in the expression levels of excitatory/inhibitory ion channels, disruption in the neurotransmitter release, changes in the axon or dendrite conductance, and other reasons. All of these changes can be caused both by genetic and environmental factors.

ENU-directed mutagenesis is a powerful tool for studying the function of mammalian genes [8,9,10]. ENU causes point mutations in a gene, often resulting in changes in the expression of entire cascades of proteins. There are several models of epileptogenesis based on pharmacological intervention. They include PTZ (Pentylenetrazole-induced seizure), Pilocarpine, and Kainate injections. Another way to cause seizures includes physical influences, such as electric shock or audiogenic induction [11,12,13,14,15]. Although these models have provided a lot of information on the genesis of seizures and helped to develop medical treatments for epilepsy, they have some limitations. Therefore, using mouse mutants, including ENU-induced ones, can help with creating models of epilepsy that might better resemble human disorders to perform more effective screening of potential antiepileptic drugs. 

Here, we report a new mutation in the mouse, *socrates* (*soc*) mapped on Chr.8, that induces audiogenic epilepsy. *Soc* mutation causes multiple changes in both the expression of ion channels and a change in the proportion of neurons and glia.

## 2. Results

### 2.1. Characterization of Epileptiform Activity and Behavior in a Novel Mouse Mutant Soc

**Figure 1:**
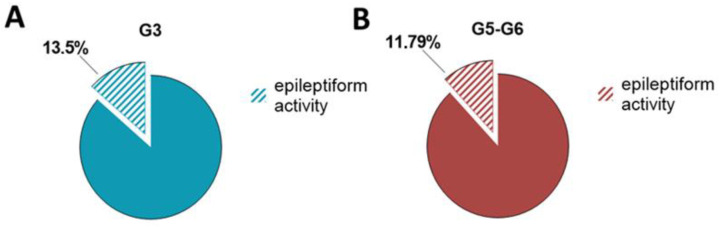
The ratio of animals with epileptiform activity among offspring of the *soc* line of different backcross generations G3 (**A**) and G5–G6 (**B**).

In order to identify novel gene alleles susceptible to audiogenic seizures, we carried out ENU-induced genetic screening [16,17]. As a result of a series of injections of ENU into C3H wild type mice, a novel mutant strain, *socrates* (*soc*), was generated. In order to map the genetic locus, the mice whose offspring showed the audiogenic seizures phenotype were backcrossed to C57Bl6 wild type mice for six generations. In G3 offspring, the manifestation of audiogenic seizures was observed in 13.5% of cases of activity in the analyzed 52 mice (Figure 1A). Analysis of the G5 and G6 descendants from subsequent crosses confirmed the presence of the trait in 11.79% of cases of epileptiform activity among the 195 mice tested (Figure 1B). 

**Figure 2:**
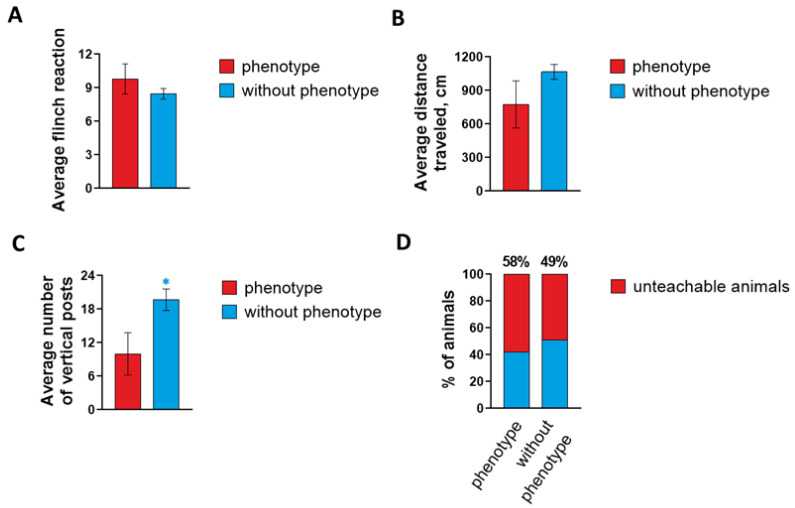
Behavioral phenotyping of *soc* mice for the acoustic startle reaction (**A**), for general motor activity in the Mouse Open Field test (**B**), and when assessing orienting–exploratory activity (**C**) and learning ability in the CPAR test (**D**). Data are presented as mean ± SD. * significance of differences between the “phenotype” group and the “without phenotype” group (*p* < 0.05, Kolmogorov–Smirnov normality test and Mann–Whitney test).

Animals were tested for the acoustic startle response. Animals that demonstrated increased excitability were considered to have a phenotype in contrast to control littermates. The numbers also correlated with the number of animals showing a positive response after audiogenic stimulation (Figure 2A). These mice were designated “phenotype” positive mice. The locomotor activity of mice of this line decreases, as evidenced by open field testing data: the average locomotor activity in individuals with the phenotype (775.14 ± 210.81 cm) is lower relative to control individuals without the phenotype (1065.41 ± 67.12 cm) (Figure 2B). The average orienting and exploratory activity in terms of the number of vertical stances of mice with the phenotype turned out to be significantly lower (10 ± 3.78 events) compared to mice without the phenotype (19.69 ± 1.92 events), which may indicate a decrease in basic activity as a result of a state of anxiety, which can be caused by increased excitability (Figure 2C). Also, based on the results of the conditioned passive avoidance reaction (CPAR) test, it was revealed that animals from the experimental group *soc* with a mutant phenotype have higher numbers of “bad learners”, because 58% of the animals did not develop a conditioned passive avoidance reflex compared to 49% of the animals without the phenotype (Figure 2D).

### 2.2. Soc Mice Show Gene Expression Differences in the Brain

**Figure 3:**
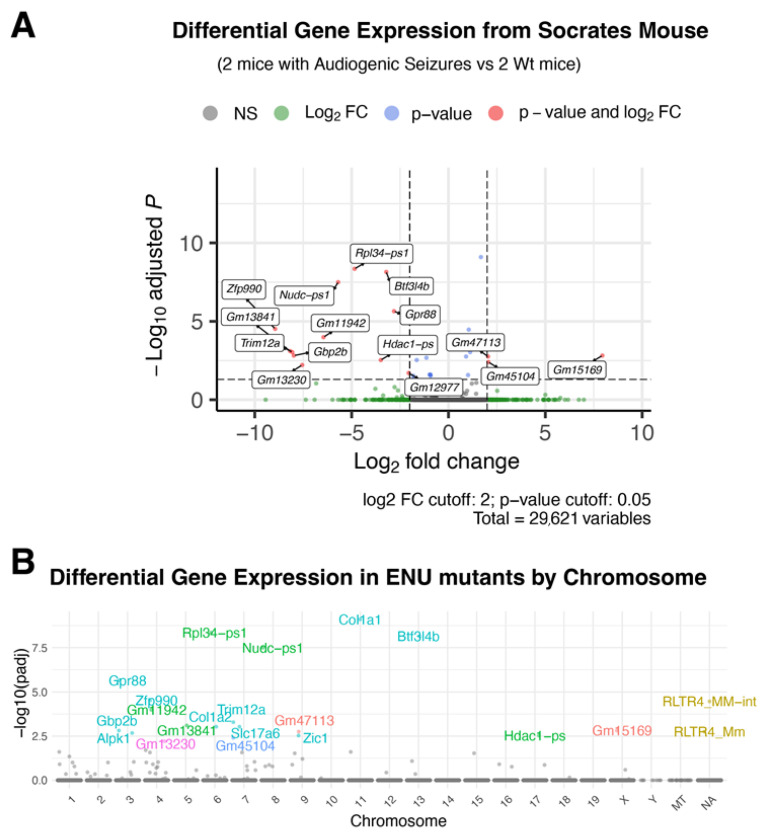
Volcano plot of genes differentially expressed in *socrates* mouse brains (**A**). Log2 Fold change is plotted on the *x*-axis and the −log10 adjusted *p* value on the *y*-axis. Points are colored according to passing adjusted *p* value or adjusted *p* value and log2Fold Change filters. (**B**) The pseudo-Manhattan plot of significantly changed genes in *soc* mouse (phenotype) by chromosome.

Using the previously developed and described SNP panel, we mapped *soc* mutation on chromosome 8 for the (8:28057473-32291828) (Figure 3B) [17]. In an attempt to identify the mutation, we carried out both full genome and Sanger sequencing of the locus. These experiments did not reveal any detectable changes in the locus. This locus is relatively gene poor, and it contains a region highly enriched in GC bases; therefore, it might not be readable by sequencing methods. Therefore, we continued phenotype analysis based on differences in seizure susceptibility and excitation level (see below). 

In an attempt to identify genes causing the phenotype, we carried out full transcriptome analysis through RNAseq. For the analysis, we isolated mRNA samples from P21 brains of the mice that were presenting the phenotype as well as from littermates that did not have seizures. These experiments led to the identification of a dozen of transcripts showing significant expression level differences between the mice with different phenotypes. Interestingly, one gene, *nudc-ps1*, was mapped on chromosome 8 within the locus that we mapped the *soc* mutation to (Figure 3A). 

As *nudc-ps1* encodes a pseudogene that is unlikely to encode a functional protein, we tested the expression of the protein-coding gene closest to the locus, *unc5D*, through in situ hybridization (ISH) analysis. Four other genes were also selected for in situ hybridization (ISH) analysis: *alpk1*, *slc17a6*, *pcp4l1*, and *zfp990.* They were selected based on either their function (ion balance for Slc17a6) or expression differences detected through RNAseq. 

**Figure 4:**
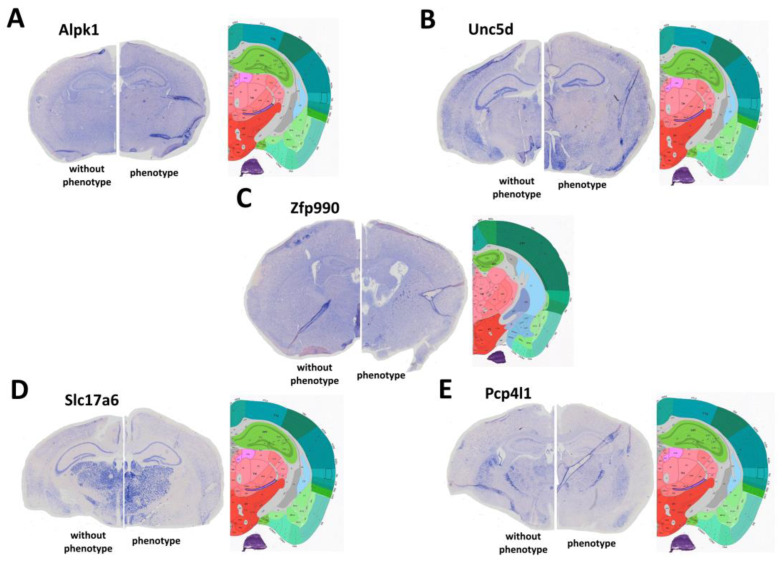
ISH with RNA probes for the genes *alpk1* (**A**), *unc5d* (**B**), *zfp990* (**C**), *slc17a6* (**D**), and *pcp4l1* (**E**) on coronal cortical sections at P21.

ISH was carried out on brain sections isolated from P21 animals (Figure 4) with or without the seizure phenotype. We found no significant differences in expression for genes *alpk1* (Figure 4A), *unc5d* (Figure 4B), or *zfp990* (Figure 4C). On the other hand, *slc17a6* (Figure 4D) and *pcp4l1* (Figure 4E) genes showed a moderate increase in expression in the cerebral cortex of animals that had seizures compared to animals that did not have them. Additionally, the *pcp4l1* gene was expressed in hippocampal cells (dentate gyrus, CA1, CA3 regions) of “without phenotype” animals, whereas the expression was downregulated in the hippocampus of animals exhibiting the phenotype.

### 2.3. Ca^2+^ Activity in Cortical Neurons of Soc Mice

**Figure 5:**
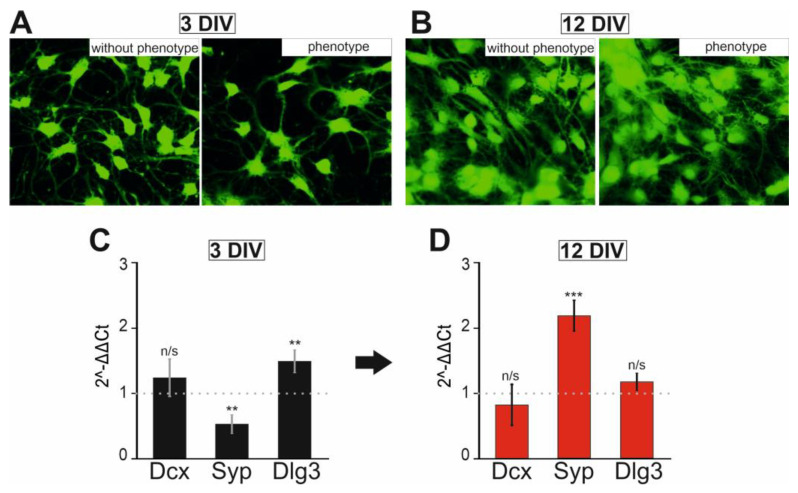
Development of the neuronal network and differentiation of neurons isolated from “without phenotype” and “phenotype” *soc* mice. (**A**,**B**) Staining of cerebral cortex cells at 3 DIV (**A**) and 12 DIV (**B**) with the Calcein probe. (**C**,**D**) Changes in the expression of genes encoding proteins that regulate neuronal differentiation at 3 DIV (**C**) and 12 DIV (**D**). n/s—data not significant (*p* > 0.05), ** *p* < 0.01, and *** *p* < 0.001.

In order to investigate the excitability of cortical neurons in vitro, we isolated them from embryonic brains of the *soc* line and measured the Ca^2+^ activity *in vitro.* They were stained with the fluorescent probe Calcein-AM on day 3 (Figure 5A) and day 12 (Figure 5B) after plating. As in these experiments we could not distinguish embryos with and without the phenotype, we measured Ca^2+^ activity independently in cells from single brains. In such experiments, we also detected two groups of samples that differed significantly. One group (ca. 40% from a litter) was showing Ca^2+^ activity levels similar to those of unrelated animals without the phenotype. The other group (ca. 60%, *soc* neurons hereafter) was showing very high excitability. As can be seen in Figure 5A,B, cell morphology and network formation occur similarly in all cultures, i.e., the number of cells in the field of view of the microscope after 3 days of cultivation is similar, and at 12 DIV, the network is fully formed due to the sprouting of neurites. Analysis of the expression of some genes encoding proteins that are markers of neuronal maturity and differentiation showed that the expression level of the *syp* gene encoding synaptophysin was significantly decreased, while *dlg3* was significantly increased (Figure 5C) in one group of neurons after three days of culture compared to another group of neurons. However, after 12 days of culture, the level of expression of the *dcx* and *dlg3* genes did not differ in neurons from both groups, and the expression of *syp* even significantly increased in the first group relative to “without phenotype” neurons (Figure 5D). These results of PCR analysis indicate that the differentiation of neurons in both experimental groups is similar. The parameters of the second group of neurons were similar to those of “without phenotype” neurons.

**Figure 6:**
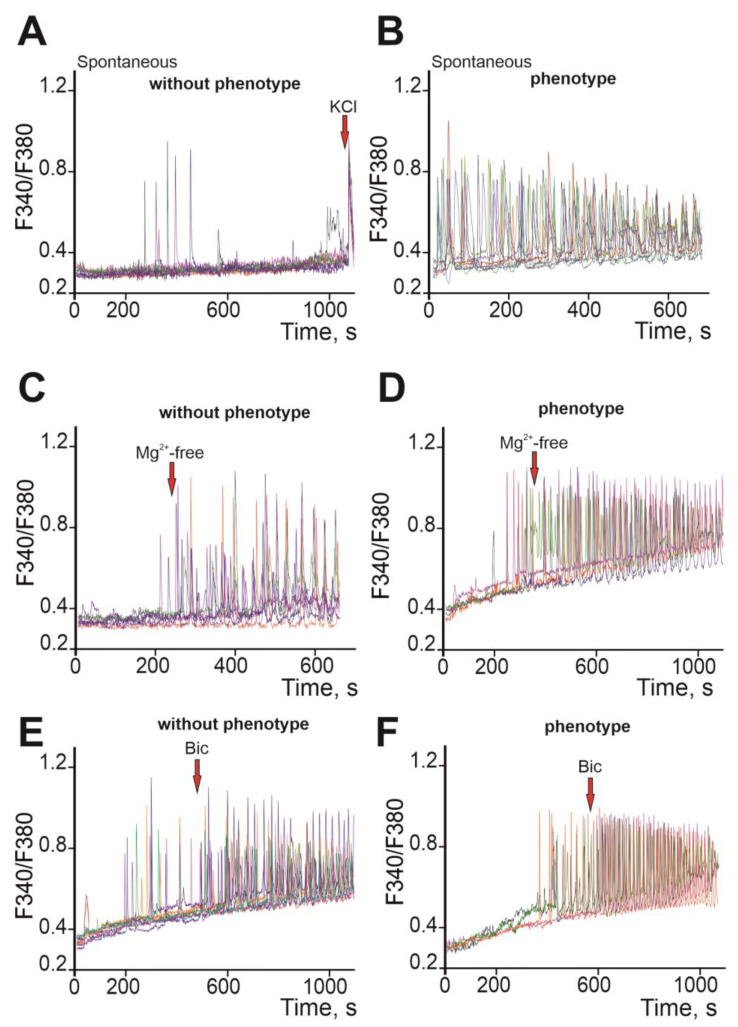
Characteristics of Ca^2+^ activity of neurons isolated from “without phenotype” and “phenotype” *soc* mice. (**A**,**B**) spontaneous Ca^2+^ signals of “without phenotype” neurons (**A**) and “phenotype” neurons (**B**). (**C**,**D**) Ca^2+^ signals of “without phenotype” (**C**) and “phenotype” neurons (**D**) when modeling epileptiform activity by excluding Mg^2+^ ions from the medium (Mg^2+^-free). (**E**,**F**) Ca^2+^ signals of “without phenotype” (**E**) and “phenotype” *soc* neurons (**F**) when modeling epileptiform activity using inhibition of GABA(A) receptors upon application of 10 μM of bicuculline. Typical Ca^2+^ signals of neurons are presented.

**Figure 7:**
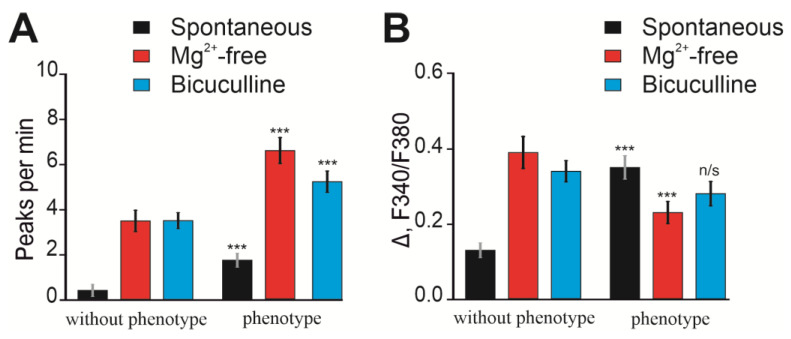
Analysis of the period (**A**) and amplitude (**B**) of Ca^2+^ impulses in “without phenotype” and “phenotype” *soc* neurons during spontaneous Ca^2+^ activity and modeling of epileptiform activity using the exclusion of Mg^2+^ ions (Mg^2+^-free) and inhibition of GABA(A)—receptors (bicuculline). Averaged results obtained on 4 cell cultures are presented. n/s—data not significant (*p* > 0.05), *** *p* < 0.001.

Thus, “phenotype” *soc* neuronal cultures do not differ in neurite network formation or differentiation from neurons isolated from “without phenotype” mice. Accordingly, a comparison of the Ca^2+^ activity of such neurons seems to be a justified approach for characterizing the *soc* mouse strain. Cortical neurons were loaded with the Ca^2+^-sensitive probe Fura-2, and spontaneous Ca^2+^ activity was recorded. In neurons obtained from the cerebral cortex of “without phenotype” mice, single spontaneous Ca^2+^ impulses were observed (Figure 6A) with a frequency of 1 ± 1 impulses per minute (Figure 7A). In “phenotype” *soc* neurons, stable high-amplitude spontaneous Ca^2+^ oscillations were recorded (Figure 6B), with an average frequency of 3 ± 2 impulses per minute (Figure 7A) and an amplitude twice as large (Figure 7B) compared to “without phenotype” neurons.

Modeling of epileptiform activity of neurons using the application of a magnesium-free medium (Mg^2+^-free) showed that in “without phenotype” neurons, the generation of asynchronous Ca^2+^ oscillations occurred (Figure 6C) with a period of 3.8 ± 2 pulses per minute (Figure 7A) and an average amplitude of 0.39 ± 0.04 (Figure 7B).

Application of Mg^2+^-free medium to *soc* neurons characterized by spontaneous Ca^2+^ activity led to the generation of Ca^2+^ oscillations (Figure 6D) with a period of 6.3 ± 2 pulses per minute (Figure 7A) and an average amplitude of 0.23 ± 0.05 (Figure 7B). 

The bicuculline model of epileptiform activity showed that the application of 10 μM of the GABA-A receptor blocker bicuculline led to the generation of high-frequency Ca^2+^ oscillations in “without phenotype” neurons occurring at an increased level of [Ca^2+^] (Figure 6E). The oscillation frequency was 3.9 ± 3 pulses per minute (Figure 7A), and the amplitude averaged 0.37 ± 0.02 (Figure 7B). Application of bicuculline to “phenotype” *soc* neurons led to an increase in the frequency of spontaneous Ca^2+^ oscillations (Figure 6F), and the frequency of bicuculline-induced oscillations averaged 5.2 ± 3 pulses per minute (Figure 7A). The amplitude of Ca^2+^ oscillations was 0.31 ± 0.05 (Figure 7B).

Thus, cortical neurons isolated from *soc* mice do not differ in network development or the expression of differentiation markers at either the early stages or at later stages in culture. At the same time, *soc* neurons are characterized by increased spontaneous Ca^2+^ activity, thus generating high-amplitude spontaneous Ca^2+^ oscillations. Modeling of epileptiform activity showed that *soc* neurons are characterized by the generation of Ca^2+^ oscillations of a higher frequency when magnesium ions are excluded or GABA-A receptors are inhibited with bicuculline, while the amplitudes of these Ca^2+^ impulses do not differ from “without phenotype” *soc* neurons. These data indicate that neurons isolated from “phenotype” *soc* mice have higher excitability.

### 2.4. Changes in the Expression Patterns of Genes That Regulate Neuronal Hyperexcitation

**Figure 8:**
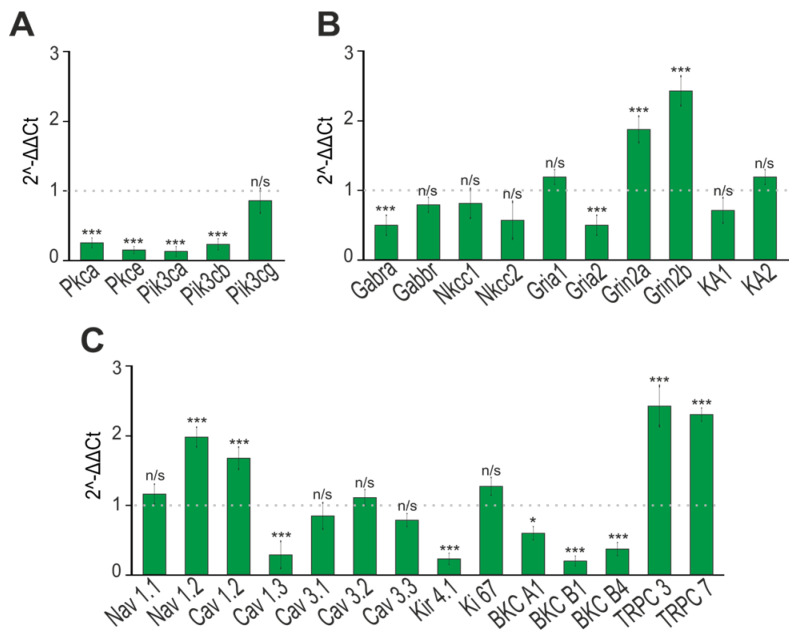
Expression of genes encoding isoforms of phosphoinositol 3-kinase and protein kinase C (**A**), inhibitory GABA receptors (GABA-A and GABA-B) (**B**), and excitatory glutamate receptors (AMPAR, NMDAR, and KAR) (**C**) in cultured cortical neurons obtained from “phenotype” *soc* mice: 1 (dashed line) is the level of gene expression in neurons obtained from “without phenotype” *soc* mice. n/s—data not significant (*p* > 0.05), * *p* < 0.05 and *** *p* < 0.001.

**Figure 9:**
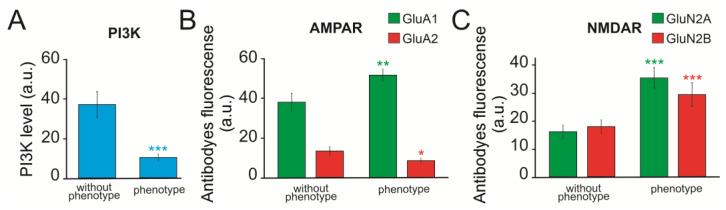
Secondary antibody fluorescence analysis reflecting phosphoinositol 3-kinase (**A**) protein levels, AMPAR (**B**), and NMDAR (**C**) subunits in “without phenotype” and “phenotype” *soc* neurons. The results presented in the figures correspond to Supplementary Figures S1 and S2. For each column, 300 ± 150 neurons were analyzed. * *p* < 0.05, ** *p* < 0.01, and *** *p* < 0.001.

The viability of neurons, their differentiation, and neurotransmission are regulated by the level of expression of genes encoding various proteins. PCR analysis of the expression of genes encoding isoforms of protein kinase C (*pkc*) and phosphoinositide 3-kinase (*pi3k*) showed that in cultured neurons isolated from the cerebral cortex of “phenotype” *soc* mice, the level of expression of these genes was significantly suppressed (Figure 8A). The results of PCR analysis were confirmed by immunocytochemical staining of neurons with antibodies against PI3K, as in “phenotype” *soc* neurons the level of PI3K protein is more than three times lower (Figure 9A) compared to “without phenotype” *soc* neurons.

The ratio of excitatory glutamate receptors and inhibitory GABA receptors might contribute to hyperexcitation and epileptiform activity [18,19,20]. In “phenotype” *soc* neurons, a decrease in the expression level of the *gabra1* (GABA-A receptor) and *gria2* (GluA2 subunit of AMPA receptors) genes, and an increase in the level of the *grin2a* and *grin2b* genes (Figure 8B), was observed. Immunocytochemical staining of neurons with antibodies against the GluA1 and GluA2 subunits of AMPA receptors showed that the level of the GluA1 subunit is significantly higher in *soc* neurons, while the content of the GluA2 subunit, on the contrary, is reduced by almost three times (Figure 9B) compared to “without phenotype” *soc* neurons. The expression level of the GluN2A and GluN2B subunits, as shown by immunocytochemical staining (Supplementary Figure S1B), was significantly higher in *soc* neurons (Figure 9C).

Various ion channels of the cytoplasmic membrane of neurons also play an important role in neurotransmission, and the disruption of their expression can lead to neuronal hyperexcitation and epileptiform activity. PCR analysis showed that in *soc* neurons, there was an increase in the expression of the *nav1.2, cav1.2, trpc3, and trpc7* genes encoding the alpha subunit of the type 2 sodium channel protein, the L-type calcium channel, TRPC3, and TRPC7 channels (Figure 8C). At the same time, in *soc* neurons, there was a decrease in the expression of the genes *cav1.3, kir4.1, bkca1, bkcb1*, and *bkcb4* encoding the alpha subunit of the L-type Ca^2+^ channel, the Kir4.1-type K^+^ channel, and calcium subunits-activated BK channels (Figure 7C).

**Figure 10:**
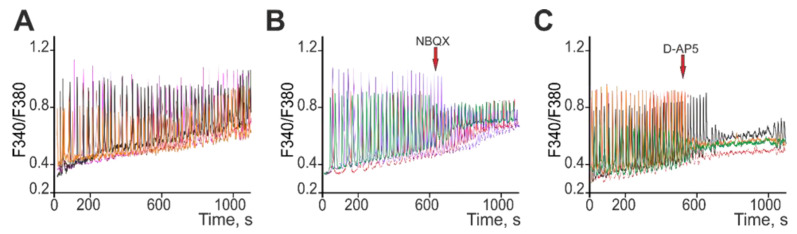
Effect of AMPAR and NMDAR blockers on spontaneous Ca^2+^ activity in neurons isolated from *soc* mice. (**A**) Spontaneous Ca^2+^ signals of *soc* neurons. (**B**) Application of the AMPAR antagonist NBQX (10 mkM) against the background of spontaneous Ca^2+^ oscillations of *soc* neurons. (**C**) Application of the NMDAR antagonist D-AP5 (50 mkM) against the background of spontaneous Ca^2+^ oscillations of *soc* neurons. Typical Ca^2+^ signals of neurons in one experiment are presented.

Because “phenotype” *soc* neurons are characterized by increased levels of expression of AMPAR and NMDAR, it can be assumed that these receptors play a leading role in the increased spontaneous Ca^2+^ activity of neurons (Figure 10A). Application of the AMPAR antagonist NBQX (10 mkM) to “phenotype” *soc* neurons with spontaneous Ca^2+^ activity led to a decrease in the amplitude of Ca^2+^ oscillations, but not their suppression (Figure 10B). At the same time, the NMDAR antagonist D-AP5 (50 µM) suppressed spontaneous Ca^2+^ oscillations (Figure 10C).

Thus, cortical neurons of *soc* mice generated by ENU-directed mutagenesis form a developed network and, in their differentiation, they do not differ from cultured neurons isolated from “without phenotype” *soc* mice. At the same time, “phenotype” *soc* neurons are characterized by increased spontaneous Ca^2+^ activity, thus generating high-frequency Ca^2+^ oscillations of a large amplitude, whereas in “without phenotype” *soc* neurons, only single Ca^2+^ impulses were recorded. When modeling epileptiform activity in vitro, it turned out that “phenotype” *soc* neurons are more prone to epileptogenesis and hyperexcitation syndrome, because in response to a magnesium-free environment and bicuculline, they generate higher-frequency and stable Ca^2+^ oscillations. The mechanism of such hyperexcitation of *soc* neurons involves an increased level of expression of NMDA receptors, a reduced level of GluA2 subunits of AMPA receptors, which are responsible for limiting the entry of Ca^2+^ ions into the cytosol, as well as a significant suppression of the expression of protein kinase C and phosphoinositol 3-kinase. It should be noted that the reduced level of expression of genes encoding neuroprotective BK channels coincides with an increased level of expression of TRPC channels of the plasma membrane, which can also contribute to the induction of epileptiform activity in “phenotype” *soc* neurons.

### 2.5. Changes in Expression Patterns of Genes That Regulate Neuronal Hyperexcitation

**Figure 11:**
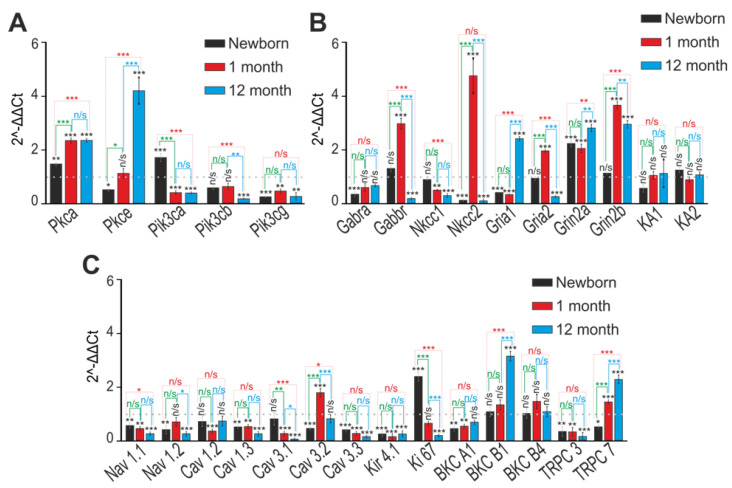
Expression patterns of genes encoding protein kinases (**A**), GABA and glutamate receptors (**B**), and membrane ion pathways (**C**) in the nucleus of the brain of newborns (black bars) of *soc* mice and during their development at 1 month (red bars) and 1 year (blue bars). For 1—dotted line, the level of expression in the brain nucleus of “without phenotype” *soc* mice is achieved. n/s—data not significant (*p* > 0.05), * *p* < 0.05, ** *p* < 0.01, and *** *p* < 0.001.

It is known that the results in in vitro models cannot always be extrapolated to in vivo models. Therefore, we tested changes in the expression patterns of genes encoding proteins involved in epileptogenesis in the cerebral cortex of mice at different ages. Total RNA was isolated from the cerebral cortex of newborn mice at 1 month and 12 months. Analysis of the expression of genes encoding isoforms of protein kinases C and PI3K (Figure 11A) showed that during maturation, the expression level of Pkca and Pkce increases and is, on average, higher in the cerebral cortex of “phenotype” *soc* mice compared to “without phenotype” *soc* mice. Then, the level of expression of genes (*pik3*) encoding PI3K is lower in both newborn and one-year-old “phenotype” *soc* mice (Figure 11A) compared to “without phenotype” *soc* mice.

In newborn mice, the level of expression of the *gabra* gene, which encodes GABA-A receptors, is significantly lower in the cerebral cortex of *soc* mice, but already at 1 month and 12 months, the expression of this gene reaches the control level (Figure 11B). The expression of the *gabbr* gene, which encodes the GABA-B receptor, in the cerebral cortex of newborn *soc* mice does not differ from “without phenotype” *soc* mice; by 1 month, its expression increased by three or more times, but by 12 months the expression decreased critically (Figure 11B). At the same time, the level of expression of the *nkcc1* gene, which encodes isoform I of the Na–K–Cl cotransporter, decreased in “phenotype” *soc* mice during maturation, and the expression of the *nkcc2* gene, which encodes isoform II of this transporter, was reduced in newborn mice and at the age of 12 months, but in 1 month, it increased five times (Figure 11B). Newborn and 1-month-old “phenotype” *soc* mice had a low level of expression of the *gria1* gene but a high level of *gria2*, which encodes the GluA1 and GluA2 subunits of AMPAR (Figure 11B). However, the expression patterns of these genes change dramatically in the cerebral cortex upon reaching the age of 1 year, when the expression of Gria1 increases but the level of Gria2 decreases (Figure 11B). As for NMDAR subunits, the cerebral cortex of “phenotype” *soc* mice is characterized by a high expression of the *grin2a* and *grin 2b* genes in all age groups (Figure 11B). The expression of the *ka1* and *ka2* genes encoding kainate receptors in the cerebral cortex of *soc* mice does not differ from “without phenotype” *soc* mice (Figure 11B).

A dramatic change was observed in the expression patterns of genes encoding ion channels in the cerebral cortex of *soc* mice. It was found that in all age groups of *soc* mice, a low level of expression of the *nav1.1, nav1.2, cav1.3, cav3.3, kir4.1*, and *trpc3* genes was observed (Figure 11C). Expression of the *ki67* gene decreased with age in *soc* mice, while the levels of *bkcb1* and *trpc7*, on the contrary, increased (Figure 11B).

Thus, *soc* mutations seem to induce disruption of the expression of several genes encoding proteins involved in the induction and regulation of epileptiform activity. There is a decreased expression of phosphoinositol 3-kinase genes, the level of which does not increase even by one year of age. The expression of most of the studied genes encoding cytoplasmic ion channels decreases during animal development, which undoubtedly contributes to the disruption of neurotransmission in the cerebral cortex of *soc* mice.

### 2.6. Soc Mutation Causes Increased Numbers of Interneurons

One of the mechanisms of epileptogenesis is a change in the excitation/inhibition balance in the neural network due to an imbalance of signaling pathways, leading to a decrease or increase in inhibition/excitation, or a combination of these factors. One of the possibilities could be a change in interneuron numbers.

**Figure 12:**
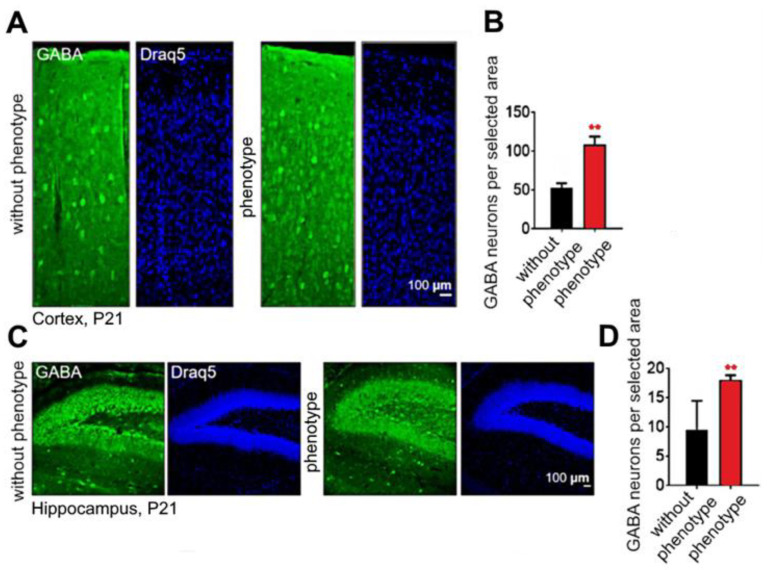
Quantification of GABA+ interneurons in the cortex (**A**,**B**) and dentate gyrus (**C**,**D**). Representative images of P21 coronal sections. Data are presented as mean ± SD. ** significance of differences in the “phenotype” group compared to the “without phenotype” group (0.001 < ** *p* < 0.01, D’Agostino–Pearson normality test and Mann–Whitney test).

To compare the number of interneurons in the brain of “without phenotype” and “phenotype” *soc* mice at P21, we carried out immunohistochemical staining using cell-type-specific markers (Figure 12A,C). In the brains of *soc* mice, we detected an increase in the number of interneurons in the cortex (“without phenotype” −52.83 ± 5.6; “phenotype” −108.0 ± 10.52 neurons/selected area) as well as in the dentate gyrus (DG) (“without phenotype” −9.5 ± 4.89; “phenotype” −18 ± 0.82 neurons/selected area) compared to control mice (Figure 12B,D). This indicates that changes in the inhibitory part of the excitatory/inhibitory balance could be one of the reasons for seizures in “phenotype” *soc* mutants [21].

**Figure 13:**
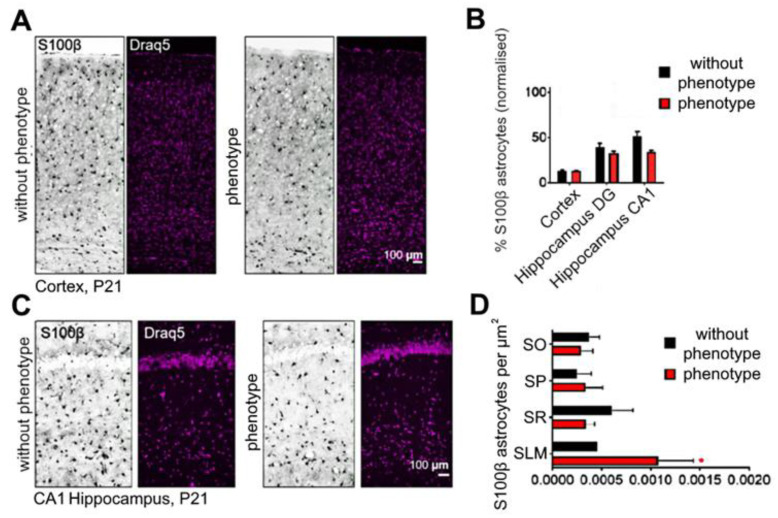
Quantification of the proportion of astrocytes in the cortex (**A**), the CA1 region of the hippocampus (**C**), considering different regions of the hippocampus (**B**) and dividing the CA1 region into zones (**D**). Representative images of P21 coronal sections. Data are presented as mean ± SD. * significance of differences between the “phenotype” and the “without phenotype” *soc* mice (0.01< * *p* < 0.05, D’Agostino–Pearson normality test and Mann–Whitney test).

Astrocytes can also contribute to epileptogenesis. They are involved in the control of ion channels, receptors, and neurotransmitter transporters, as well as the control of K^+^ concentration. Therefore, we tested the number of astrocytes in *soc* brains. We could show that the number of astrocytes in the cortex (Figure 13A) and hippocampus (Figure 13B,C) in the “phenotype” group relative to the “without phenotype” group is not statistically different (Cortex: “without phenotype” −13.17 ± 0.88%; “phenotype” −13.26 ± 0.08%; Dentate gyrus: “without phenotype” −39.42 ± 4.23%; “phenotype” −32.56 ± 2.28%; CA1: “without phenotype” −51.23 ± 5.38%; “phenotype” −33.75 ± 2.10%) (Figure 13A). We also divided the CA1 region of the hippocampus into zones (stratum orientalis (SO), stratum pyramidal (SP), stratum radiatum (SR), and stratum lacunosa molecular (SLM)) and analyzed the number of astrocytes (Figure 13D). (SO: “without phenotype” −0.00037 ± 0.0001, “phenotype” −0.0002 ± 0.0001 SP: “without phenotype” −0.0002 ± 0.0001, “phenotype” −0.0003 ± 0.0002; SR: “without phenotype” −0.0006 ± 0.0002, “phenotype” −0.0003 ± 0.00001; SLM: “without phenotype” −0.0005 ± 0.00001, “phenotype” −0.0011 ± 0.0004 astrocytes/µm^2^). In the SLM zone, *soc* mutants showed a significant increase in the number of astrocytes (Figure 13D). The detected gliosis in mice of the “phenotype” group can affect the state of the excitation/inhibition balance [21].

## 3. Discussion

Over the past three decades, knockout mice have been widely utilized as valuable models for studying various human diseases and providing insights into the functions of individual genes. However, they may not always be optimal for studying certain aspects of human disease pathogenesis and specific gene functions. This is due to the fact that complete loss of a gene can lead to embryonic mortality or more severe phenotypes compared to patients with single nucleotide mutations. Knockouts often exhibit extremely severe phenotypes and may trigger compensatory mechanisms unrelated to neurological diseases [22]. It is widely agreed upon that most human diseases are caused by mutations that primarily affect protein structure, and thereby binding affinity, or disrupt protein function in a subtler manner than complete inactivation [23,24]. Additionally, it is increasingly recognized that genetic variants, such as single nucleotide polymorphisms (SNPs), outside of protein-coding regions likely play a significant role in disease development [25]. Therefore, alternative mouse models are crucial, and random mutagenesis has become an established tool for elucidating gene function and investigating novel disease pathways [26]. Several mouse strains with impaired brain function have already been generated using ENU-directed mutagenesis. For example, *foxp2* mice (*s321x/s321x* gene) exhibit severe motor impairment, developmental delay, and cerebellar foliation defects, and they survive for 3–4 weeks [27]. *gars* mice (*c201r/c201r* gene) experience neurodevelopmental delay and survive for 3 weeks [28]. *tuba1a* mice (*d85g/+* gene) display hyperactivity and neuronal migration defects [29]. The *disc1-q31l* homozygous mutant exhibits depressive-like behavior, while the *disc1-l100p* homozygous line displays abnormalities related to schizophrenia [30]. Preliminary data from an ENU screen for modifiers of *k3* mice, an Alzheimer’s disease (AD) model expressing mutant Tau protein, have recently been reported [31]. This model has been extensively studied to understand pathological and behavioral abnormalities associated with AD [32]. In terms of epilepsy, a *myk* mice strain was obtained using ENU-directed mutagenesis. The dominant *myshkin (myk)* mutant was selected based on its small size and the presence of spontaneous, recurrent, convulsive seizures, establishing it as a model for epilepsy [33]. In the *myk* brain, a missense mutation in the Na^+^, K^+^-ATPase (NKA) a3 isoform (*atp1a3*) leads to an approximate 40% reduction in enzyme activity. This gene has been implicated in bipolar disorder and rapid-onset dystonia–parkinsonism [34,35]. In this study, we successfully generated and characterized a new strain of mice with a high susceptibility to epilepsy. Furthermore, we investigated the signaling pathways disrupted by this mutation. 

Many animal models of epilepsy are now available for specific research purposes [36,37,38]. Mouse models of monogenic epilepsy have played and will continue to play a crucial role in advancing our understanding of the underlying mechanisms of this condition. Their translational relevance to human clinical research and practice can be emphasized in several ways. Firstly, these models provide valuable information about the mechanisms and pathways involved in the disease, which serves as a foundation for comprehending the disease’s mechanisms in humans and facilitates the development of targeted therapies. Secondly, mouse models can be utilized to assess the efficacy and safety of potential treatments for monogenic epilepsy, thus aiding in the discovery of effective therapeutic interventions [39]. Furthermore, mouse models allow for the identification and validation of potential biomarkers associated with monogenic epilepsy, which can be instrumental in diagnosing the condition, predicting its progression, and monitoring an individual’s response to treatment [40]. Lastly, mouse models provide a preclinical testing platform for evaluating novel therapies, such as gene therapy, targeted drugs, and neurostimulation techniques, before their translation into clinical trials [41]. These avenues highlight the significant role of mouse models in furthering our knowledge and improving therapeutic approaches in the field of monogenic epilepsy.

It is now generally accepted that the development of neurological diseases is determined by interactions between genes and the environment, where changes in gene expression occur under different conditions but the DNA sequence is maintained. The main epigenetic mediators can be considered histone modification, DNA methylation, and micro RNA [42]. Knowledge of the genes encoding proteins involved in epilepsy allows us to formulate adequate treatment for patients. The presented study carried out a comprehensive analysis of the expression of genes encoding proteins involved in epileptogenesis. Cultivation of cortical *soc* neurons showed that they do not lag behind WT neurons in development or network formation, as indicated by the staining of neurites on days 3 and 12 of cultivation. In addition, genes encoding markers of neuronal differentiation are expressed in *soc* neurons at the level of WT neurons or even higher. It is known that the expression of Doublecortin (*dcx* gene), PSD95 (*dlg4* gene), and Synaptophysin (*syp* gene) is enhanced during the development of neuronal cultures [43,44]. 

An imbalance between excitation and inhibition in the brain may be the cause of epilepsy [45]. The main role in the process of neuronal network excitation is assigned to glutamate, which transmits excitation from one neuron to another. An increase in the glutamate concentration in the extracellular space has been shown during epileptic seizures [46,47], and glutamate receptor agonists can enhance seizures in humans and animals [48,49]. Glutamate receptor antagonists are able to suppress seizures. Remacemide, a mild inhibitor of NMDA receptors, showed effectiveness when used as additional therapy in patients with focal seizures. However, when used as a standalone treatment, remacemide was found to be significantly less effective than carbamazepine for patients with focal seizures [50]. On the contrary, perampanel, an AMPA antagonist, has been approved as an anti-seizure medication for both focal seizures and primary generalized tonic–clonic seizures [51]. The *GRIN1* and *GRIN2A* genes, which encode the NR1 and NR2 subunits of NMDAR, are of particular importance in the development of epileptogenesis. *GRIN1* (Glutamate ionotropic receptor NMDA type subunit 1) is a gene located on chromosome 9, which encodes the NR1 subunit of the NMDA receptor (which determines neuronal excitability) and is involved in synaptic plasticity, the response to stress, and the pathogenesis of several neuropsychiatric diseases [48,52]. The *GRIN2A* gene (Glutamate ionotropic receptor NMDA type subunit 2A) encodes the NR2A subunit of the NMDA receptor. The NR2 subunit regulates the release of glutamate into the synaptic cleft and controls the activity of ion channels; it also plays an important role in normal neuronal development, synaptic plasticity, and memory [52,53]. It has been shown that disturbances in the expression of the *GRIN1* gene are associated with the development of early forms of epileptic encephalopathies, schizophrenia, and mental retardation [54]. Based on the results of experimental studies, it has been established that changes in the *GRIN2A* gene are the main genetic risk factor for various types of genetic (idiopathic) epilepsy [55,56]. Mutations of the *GRIN2A* gene are rare in benign epileptiform sleep disorders [55]. Also, mutations of the *GRIN2A* gene have been identified in epilepsy with electrical status epilepticus of slow-wave sleep, epilepsy syndromes associated with ESES syndrome, Landau–Kleffner syndrome [55,57], and in autosomal-dominant Rolandic epilepsy [58]. In 17.6% of children with epileptic encephalopathy with continued spike-wave activity during sleep, a mutation of the *GRIN2A* gene, which encodes the alpha subunit of NMDA receptors, was identified [59]. Activation of NMDAR allows the influx of sodium ions and the outflow of potassium ions, which ensures depolarization of the neuronal membrane. It has been shown that in foci of focal cortical dysplasia characterized by high epileptogenicity, NMDAR expression increases [60]. In addition, pathological activation of NMDAR leads to hyperexcitation and increased activity of neurons, which is the pathophysiological basis for epileptogenesis [61,62]. Our studies have shown that the expression level of genes encoding NMDAR subunits is increased both in the cell culture of *soc* cortical neurons and in the cerebral cortex of mice of different ages (newborns and 1 and 12 months of age). The results of PCR analysis are confirmed by staining cell cultures with antibodies against the GluN2A and GluN2B subunits of NMDAR. Abnormalities in the expression of the *GRIN2* gene in monogenic forms of epilepsy have been identified; however, NMDA receptors are widely expressed in the brain and play a critical role in excitatory neurotransmission, and epilepsy in such patients is only one of the symptoms along with movement pathologies, cognitive impairment, and severe forms of autism [63,64]. Signs of severe epilepsy have also been identified in Mowat–Wilson syndrome, which is caused by a violation of the expression of the *SIP1* gene [65], Rett syndrome, which is caused by a violation of *MECP2* [66], and Satb2-associated syndrome, which occurs when the expression of the transcription factor *SATB2* is disrupted [67]. In all of these diseases, there is increased excitability of neuronal networks and abnormal Ca^2+^ activity of neurons and even astrocytes, disturbances in the expression patterns of a large number of genes involved in neurotransmission, and a tendency of brain cells to damage and death under certain loads exceeding physiological ones, when “normal” cells easily cope with external influences [68,69,70]. Similarly, neurons derived from “phenotype” *soc* mice are characterized by increased spontaneous Ca^2+^ activity and differ from “without phenotype” *soc* neurons by increased frequency and amplitude of Ca^2+^ oscillations in bicuculline and Mg^2+^-free models of epileptogenesis.

The expression of AMPARs is increased in cases of increased neuronal excitability following epilepsy [71]. Studies have shown that the usage of NBQX, a competitive antagonist that targets AMPARs, can prevent the development of hypoxia-induced spontaneous recurrent seizures in neonatal rats [72]. Additionally, it has been found to suppress focal electrographic seizures in mice with KA-induced epilepsy [73] and to reduce the onset of seizures induced by pentylenetetrazole (PTZ) in adult rats [74]. Similar changes in the subunit composition of AMPARs observed in animal models have been found in individuals with epilepsy. For instance, a decrease in the GluA2 subunit and an increase in the GluA1/GluA2 ratio have been observed in brain tissue from epilepsy patients [75], suggesting that these alterations could potentially contribute to the occurrence of persistent seizures. These results correspond well with our data, which were obtained from differentiated cortical neurons in culture, when the level of expression of the GluA2 subunit was reduced, and the expression of GluA1, on the contrary, was at an increased level relative to WT neurons. At the same time, in newborn “phenotype” *soc* mice and at the age of 1 month, the expression of the *gria1* and *gria1* genes encoding these subunits was reduced. At the same time, in one-year-old “without phenotype” *soc* mice, a trend similar to cell cultures was observed. At the same time, the inhibitory analysis data showed that the AMPAR antagonist did not affect the generation of spontaneous Ca^2+^ oscillations of “phenotype” *soc* neurons but rather reduced their amplitude.

There are limited clinical data available regarding the involvement of KARs (kainate receptors) in the development of epilepsy. However, animal models using kainate-induced status epilepticus have provided significant insights [76,77]. During this process, excitotoxic neuronal death occurs. Although postsynaptic KARs only mediate a small fraction of ionotropic synaptic neurotransmission, they play a crucial role in synaptic integration and the establishment, maintenance, and regulation of neural circuits [78,79]. Studies have demonstrated that selective activation of GluK1-containing KARs, which are primarily found in GABAergic interneurons [80], can trigger seizure discharges [81]. Additionally, GluK2-containing KARs in glutamatergic principal neurons have central roles in seizure generation and pathogenesis [82]. *Gluk2* knockout mice exhibit reduced susceptibility to seizures induced by kainate injection [83], and selective elimination of KARs specifically at CA3 synapses mitigates kainate-induced seizures [84]. Furthermore, neonatal mice lacking GluK2-containing KARs are also less prone to hypoxic seizures [85]. It is believed that the activation of presynaptic GluK1-containing KARs suppresses GABA release, thereby reducing inhibition. Simultaneously, activation of postsynaptic GluK2-containing KARs increases excitability in principal neurons. However, no changes in expression patterns were found in *soc* mice of different age groups or in cultured cortical neurons. All of the above concerns the excitatory component of neurotransmission. Regarding inhibition, *soc* mutation seems to have disrupted the expression of genes encoding GABA receptors in *soc* mice. It was found that in cultured *soc* mice neurons, there is a decrease in the expression of GABA-A receptors, which occurs against the background of an increase in the number of excitatory glutamate NMDA receptors. This is also associated with the increased Ca^2+^ activity of these neurons in a model of epileptogenesis when GABA(A) receptors are blocked with bicuculline. The results obtained from cerebral cortex tissue showed that the level of expression of the *GABRA* gene, which encodes GABA(A) receptors, was reduced in newborn “phenotype” *soc* mice, and during adulthood, the level of this gene did not differ from “without phenotype” *soc* mice. As for GABA(B) receptors, their expression level did not change in cell culture; in the cortex tissue, they significantly increased at the age of 1 month, but in one-year-old mice, the expression level of GABA(B) receptors decreased dramatically. All of these changes undoubtedly play an important role in the induction of epileptogenesis. It has been shown that defects in the gene encoding GABA receptors, as well as disturbances in their expression, lead to the development of epilepsy in children [86,87]. For example, disturbances in the expression of the *GABRA* gene, which encodes the GABA-A receptor, accompany idiopathic generalized forms of epilepsy, and disturbances in the *GABBR* gene, which encodes the GABA-B receptor, are found in focal forms of epilepsy [88,89]. At the same time, synapses containing inhibitory GABA receptors are characterized by less pronounced neuroplasticity compared to excitatory glutamate receptors, but they are responsible for the important process of suppressing seizure activity in the brain [90]. Therefore, under conditions of increased activity, GABAergic neurons die and increased excitation occurs [91,92]. 

Interestingly, we obtained results demonstrating *soc*-mutation-dependent changes in the expression of genes encoding channel proteins in the membrane of neurons. In a mature cell culture of *soc* neurons, the expression of predominant channels (the alpha subunit of the L-type Ca^2+^ channel, the Kir4.1-type K^+^ channel, and subunits of calcium-activated BK channels) was associated with the transport of Ca^2+^ ions or activated Ca^2+^ ions. At the same time, there was an increase in the expression of several sodium channels and TRPC channels, which are strictly involved in the genesis of epilepsy. Also, in the cortex, *soc* mutation led to a general suppression of the expression of genes encoding almost all of the studied channels, except TRPC, which increased with the age of the animals. Missense mutations of the *SCN8A* (sodium voltage-gated channel alpha subunit) gene lead to disruption of the functioning of sodium channels and the development of severe pediatric drug-resistant epileptic encephalopathy. However, the sodium channel blocker phenytoin was quite effective in controlling epileptic seizures [93]. Epileptiform activity in Dravet syndrome, caused by impaired expression of the *SCN1A* gene (alpha subunit of sodium channel protein type 1), can be stopped with high efficiency by stiripentol. Meanwhile, sodium channel blockers in this syndrome can increase epileptic seizures [89,94]. Disturbances in the expression of sodium and potassium channels in the plasma membranes of neurons are also closely associated with the development of epilepsy. It has been shown that disturbances in the expression of the *SCN2A* gene, which encodes the alpha2 subunit of sodium channels, are associated with the development of benign familial epilepsy of infancy and epileptic encephalopathy [94]. Mutations in genes encoding neuronal ion channels are known to lead to the development of epilepsy. Mutations in the *KCNQ2* gene, which encodes a potassium channel crucial for neuronal excitability, have been linked to a specific type of epilepsy called Benign Familial Neonatal Epilepsy (BFNE). These genetic variations can interfere with the normal functioning of the potassium channel, thus resulting in altered neuronal activity and the occurrence of seizures [95,96]. Disruption of *KCNT1* (Potassium Sodium-Activated Channel Subfamily T Member1) gene expression causes epilepsy in infants with migratory focal seizures. Once the genetic cause of such epilepsy is clearly established, it is possible to practically inhibit the symptoms of epilepsy and improve psychomotor development with the help of quinidine [97,98]. It is likely that both an increase in the expression of membrane channels and a massive suppression of their expression disrupt neurotransmission and contribute to the development of epilepsy. At the same time, more detailed studies of the fine regulatory mechanisms of changes in the expression of these channels are necessary, and the presented data are an important observation accompanying ENU-directed mutagenesis in the cerebral cortex. Unlike monogenic epilepsy, multifactorial epilepsies exhibit a complex inheritance pattern characterized by the involvement of multiple genes and environmental factors. The precise genes associated with multifactorial epilepsies remain elusive due to the contribution of numerous genetic variations to the overall risk. However, research suggests that a combination of genetic factors, including ion channels, neurotransmitters, and genes related to neuronal development, potentially play a role in the development of multifactorial epilepsies.

Our findings indicate that *soc* mutation resulted in the suppression of the expression of key protein kinases: protein kinase A (PKA) and phosphoinositol 3-kinase (PI3K). It was found that in cultured “phenotype” *soc* neurons, there is a decrease in the expression of genes encoding all isoforms of these kinases. In the cortex tissue, the level of PKA expression decreased only in newborn “phenotype” *soc* mice, and at 1 and 24 months, the expression level of this kinase was higher compared to the cortex tissue of “without phenotype” *soc* mice. However, the expression level of genes encoding PI3K isoforms decreased in the cortex of “phenotype” *soc* mice of all ages. While the target of rapamycin (mTOR) has been shown to be involved in epileptogenesis [99,100], the role of PI3K in seizure activity has only become apparent in recent years. 

Mutations in the PI3K-AKT-MTOR signaling pathway give rise to developmental brain overgrowth syndromes that have significant clinical implications. Individuals affected by these mutations exhibit a range of phenotypes, including dysplastic megalencephaly, hemimegalencephaly, and focal cortical dysplasia. They also commonly experience comorbidities like hydrocephalus, autism, and intellectual disability [101]. Additionally, these mutations are responsible for causing focal epilepsy, which accounts for approximately 25–50% of all cases of intractable epilepsy in children [101,102]. PI3K inhibition has been shown to occur in an organotypic model of epileptogenesis in the hippocampus [103], seizures associated with brain overgrowth disorders [104], and electroconvulsive seizures [105]. A decrease in PI3K phosphorylation was found upon administration of kainic acid [106]. It has been shown that the expression of the PI3K protein is significantly lower in mice with epilepsy [107], which corresponds well with our results. It is known that apoptosis plays an important role in the death of cerebral neurons after epilepsy, and the expression and activity of PI3K contribute to the protection of neurons from apoptosis caused by epilepsy [108]. On the one hand, it has been shown that in the Nestin-cre, Pik3caE545K mouse model of epileptogenesis, overactivation of Pik3ca causes disturbances in the functioning of neurons and leads to hyperexcitation [109]. On the other hand, deletion of the transcription factor Satb1 in cortical neurons leads to neuronal hyperexcitation and correlates with the suppression of PI3K expression [110]. Our observations of ENU-induced changes in the kinome do not answer the question of the role of PI3K in epileptogenesis, and they require detailed study using additional methods, but they are a clear characteristic of the *soc* mutant mouse strain.

## 4. Materials and Methods

### 4.1. Generation of the Soc Mouse Strain Using ENU-Directed Mutagenesis and Breeding Scheme—Identifying Signs of Epilepsy

**Figure 14:**
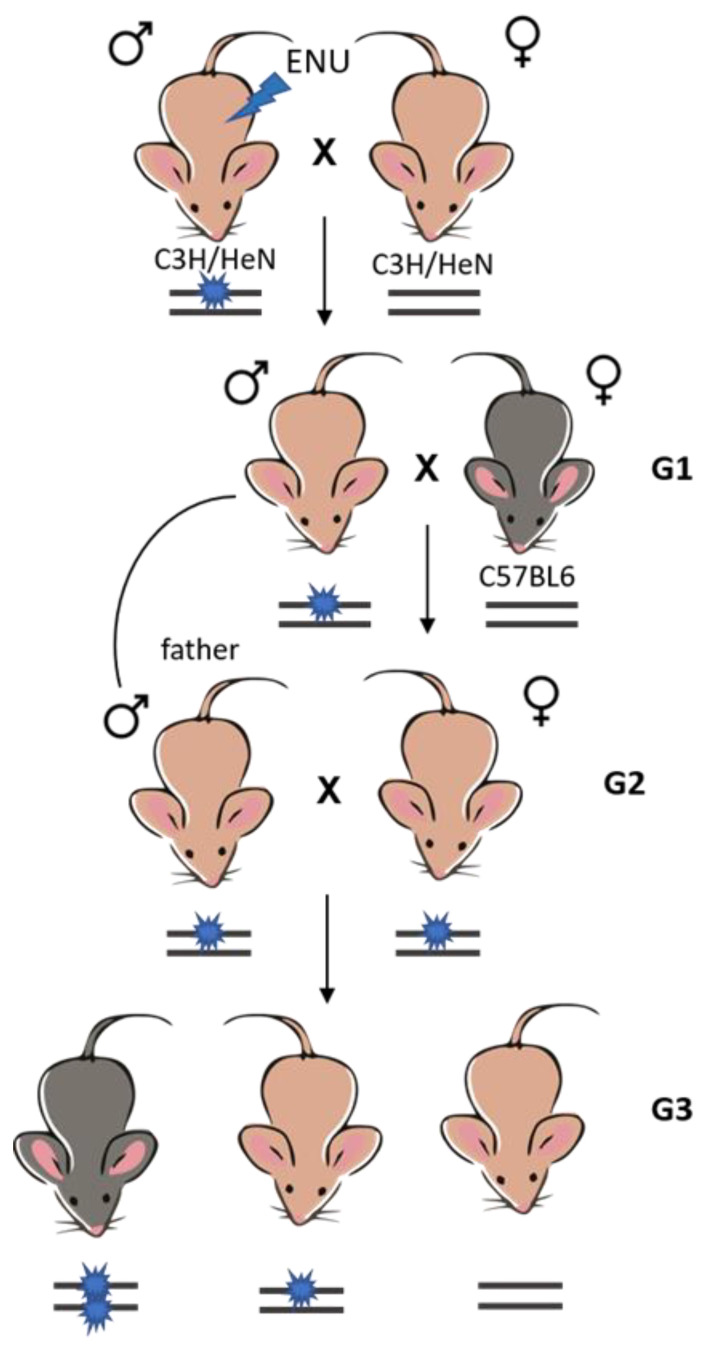
Crossing scheme with backcross stage employed.

The ENU (Sigma-Aldrich, Burlington, MA, USA) treatment was performed as previously described (Borisova et al., 2018). Briefly, 80 μg/kg of ENU was administered intraperitoneally to 8-week-old C3H/HeN mice. As a rule, the period of sterility after 3 times administration of ENU is 10–15 weeks. Males subjected to ENU mutagenesis, which restored their fertility, were crossed with intact C3H/HeN females to obtain the first generation (G1) of offspring. The generation of G1 males obtained from them was subsequently crossed with C57Bl/6 females to obtain genetic polymorphism. Next, G2 females obtained as a result of the previous crossing were mated with their G1 fathers to increase the likelihood of detecting mutations, and G3 offspring were obtained from them, in which fixed recessive mutations were searched (Figure 14).

To consolidate the resulting mutations in the *soc* strain, the following crossing techniques were used: G3 females/males were crossed with G1 individuals; G3 males and females without/with a phenotype were backcrossed to their G2 parents; and G3 males with/without a mutant phenotype were also crossed.

To select G3 generation mice for the presence of a mutant epileptic phenotype after ENU-directed mutagenesis, one of the generally accepted and widely used models of seizure activity, audiogenic stimulation, was chosen. The setup for generating audiogenic seizures in mice was created based on the Startle and Fear condition setup (PanLab, Barcelona, Spain; Stoelting, Wood Dale, IL, USA). Mice were observed using a LifeCam Cinema HD video camera (Microsoft, Redmond, WA, USA). Testing of mice was carried out at the age of 21–25 days after birth (P21–P25), and it is described in more detail in a previous study [16,17]. Briefly, mice were presented with a single 110 dB sound from an electromechanical bell. The sound stimulus was stopped immediately after the onset of audiogenic seizures or after 60 s if there was no response. The degree of manifestation of convulsive activity was assessed using the modified Krushinsky scale [111].

### 4.2. Basic Behavioral Phenotyping

In order to compare the phenotypic parameters of both animal groups (individuals of the *soc* line with an epileptic phenotype and individuals of the same line without the mutant trait), basic behavioral phenotyping was used, which involves testing for motor reactions and analyzing orienting–exploratory and learning ability. A specialized test was also conducted for rodents with a predisposition to audiogenic epilepsy. For this purpose, protocols, such as measuring the acoustic startle response, the Mouse Open Field test, recording the number of vertical stands, and testing the conditioned passive avoidance reflex (CPAR), were implemented.

#### 4.2.1. Acoustic Startle Response

Analysis of the acoustic startle response was carried out with the help of a setup for measuring the startle reflex from Packwin (Panlab, Spain), equipped with a module for delivering sound signals against a background of white noise (60 dB). The mouse is placed in the installation in a fixation box, and the animal’s flinch is recorded by the change in the force of pressure on the grid under it. The experimental protocol implemented was:

Adjustment period—7 min;
Stimulus (110 dB)—10 times;
Prestimulus-1 (70 dB)—10 times;
Prestimulus-2 (80 dB)—10 times;
Prestimulus-3 (85 dB)—10 times;
Prestimulus-4 (90 dB)—10 times;
No sound stimulus—10 times;
Signals are sent in randomly at an interval of 100 ms 10 times.

#### 4.2.2. Mouse Open Field Test and Recording the Number of Vertical Stands

The installation used is a square arena with a brightly lit area (45 × 45 × 45 cm^3^) (Harvard Apparatus, Barcelona, Spain). The adaptation period is 20 min, and then the study takes place in the installation for 5 min. ActiTrack software (V2.7) records the following parameters:

- Total mileage, including mileage in the central sector and the periphery (in cm);
- Number of vertical stands.

#### 4.2.3. Conditioned Passive Avoidance Response

For the experiment, a chamber (25 × 25 × 25 cm^3^) with an electrified lattice floor is used, and it is divided by a partition with a hole into two identical compartments—darkened and illuminated (Panlab, Spain). During training, the mouse is placed once in the light compartment of the chamber with its back directed to the dark compartment. The latent period (LP) of stay in the light compartment of the chamber is recorded. When moving into the dark compartment of the chamber, the mouse receives electrodermal stimulation on its paws (current 0.45 mA) for 5 s. The experiment is repeated on the 2nd day.

### 4.3. Cortical Neuroglial Culture Preparation

The process of mixed neuroglial cell cultures preparation, which was previously described in detail [68,69,70], was followed. The procedure for isolating and culturing neurons was identical for the cortex of *soc* mutant mice with phenotype and without phenotype. To minimize variation in gene expression and signaling system activity between individual mice, the cortex of a single mouse was used to obtain ten Petri dishes with cell cultures. In brief, newborn puppies that were between 0 and 1 day old were euthanized using an overdose of halothane and decapitated. The cerebellar cortex of the mouse was then excised with ultra-fine forceps, placed in a test tube, and incubated for 2 min, and the supernatant was removed using a pipette. The cells were treated with 2 mL of trypsin (0.1% in Ca^2+^- and Mg^2+^-free Versene solution, SAFC, Taufkirchen, Germany, Cat. #59427C) and incubated at 37 °C with shaking at 600 rpm for 10 min. The trypsin was inactivated with an equal volume of cold embryo serum. Next, the samples were centrifuged at 300× *g* for 5 min. The supernatant was removed, and the cells were washed twice with Neurobasal A medium (Thermo Fisher Scientific, Waltham, MA, USA, Cat. #10888022). Before plating, the cells were resuspended in Neurobasal-A medium containing 0.5 mM glutamine (Sigma-Aldrich, St. Louis, MI, USA, Cat. #G7513), 2% B-27 (Thermo Fisher Scientific, Waltham, MA, USA, RRID: CVCL_A315), and gentamicin (20 μg/mL, Sigma-Aldrich, St. Louis, MI, USA, Cat. #G1397). Before planting the culture, coverslips of 25 mm diameter with 6 mm diameter glass rings positioned on them (VWR International, Radnor, PA, USA, Cat. no. 48382-085) were coated with poly-L-lysine. In total, 200 µL of cell suspension was placed directly into the glass circles. The culture was incubated for five hours in an incubator at 37 °C and 5% CO_2_ before the glass rings were removed. Thereafter, the culture was incubated under the same conditions, and two thirds of the culture medium was replaced every three days with a fresh medium. Experiments were conducted on days 10 and 14 of in vitro cultivation (DIV).

### 4.4. Immunocytochemical Method

To detect AMPAR and NMDAR subunits, as well as PI3K levels in neurons, we utilized an immunocytochemical assay. The cells were fixed with a solution of 4% paraformaldehyde and 0.25% glutaraldehyde in 1x PBS for 20 min, followed by three ice-cold 1x PBS washes lasting 5 min each. The addition of glutaraldehyde to the fixative solution was performed to minimize the loss of BDNF during cell permeabilization. To permeabilize the cells, a solution of 0.1% Triton X-100 was applied for 15 min. The fixed cells were then incubated with 10% donkey serum for 30 min at room temperature to block non-specific antibody binding sites. Afterward, the cells were incubated overnight at 4 °C with primary antibodies targeting the proteins of interest. Following three 5 min washes with PBS, the cells were probed with secondary antibodies conjugated with fluorescent labels. The antibodies used in this study included: rabbit polyclonal anti-NMDAR2B antibody (Invitrogen, Waltham, MA, USA, Cat. #PIPA585632), mouse monoclonal anti-NMDAR2A antibody (Abcam, Cambridge, UK, ab240884), GluR1 polyclonal antibody (Thermo Fisher Scientific, Waltham, MA, USA, Cat. #PA5-95207), GluR2 monoclonal antibody (6C4) (Thermo Fisher Scientific, Waltham, MA, USA, Cat. #32-0300), purified rabbit monoclonal antibody to PI3-Kinase p85 alpha (Abcam, Cambridge, UK, EPR18702, ab191606), donkey polyclonal secondary antibody to rabbit IgG (H+L) labeled with Alexa Fluor-647 (Jackson ImmunoResearch Europe LTD, Cambridge, UK, RRID: AB_2492288), donkey anti-rabbit IgG (H+L) highly cross-adsorbed secondary antibody labeled with Alexa Fluor-488 (Thermo Fisher Scientific, Waltham, MA, USA, Cat. #A-21206), and donkey polyclonal secondary antibody to mouse IgG—H&L labeled with Alexa Fluor-594 (Abcam, Cambridge, UK, RRID: AB_2732073). The dilutions of primary and secondary antibodies were prepared according to the manufacturer’s recommendations for immunocytochemical staining. The fluorescence signals from the antibodies were visualized using an inverted confocal microscope (Leica TCS SP5, Wetzlar, Germany). The fluorescence of the secondary antibodies was recorded using the same microscope settings for both control and experimental cell culture groups. Fluorescence analysis was performed using ImageJ 2002 software (RRID: SCR_003070) with the assistance of the Analyze particles and Time series analyzer plugins. The detailed immunocytochemical staining technique is shown in our previous works [112,113].

### 4.5. Immunohistochemistry

Immunohistochemical analysis was conducted on brain samples taken from S8-3 mice at P21. The brains were fixed through intracardiac perfusion using a solution of 4% PFA/PBS. Following fixation, the brains were incubated sequentially in 15% and 30% sucrose. Coronal cryosections, with a thickness range of 12–16 µm, were then prepared using a Leica TCS SP5 cryotome from Leica, Germany. The samples were incubated in a blocking solution composed of 10% horse serum from Gibco, Thermo Fisher Scientific, Waltham, MA, USA, along with 0.05% TritonX-100 sourced from Roche, Switzerland, in 1× PBS for 30 min. For primary antibodies, Rabbit Anti-GABA 1:300 (Abcam, Cambridge, UK) and Rabbit anti-S100beta 1:300 (Abcam, Cambridge, UK) were utilized. Sections were incubated in a primary antibody’s solution overnight at 4 °C. Subsequently, the samples were incubated with secondary antibodies (namely, Donkey anti-rabbit AlexaFluor Cy3 (1:300, Jackson Immunoresearch, Cambridge, UK) and Donkey anti-rabbit (H+L)-HRPO (1:1000, Jackson Immunoresearch, UK)), along with a nuclear dye, Draq5 (1:2000, Thermo Fisher Scientific, Waltham, MA, USA). Visualization of the resulting immunohistochemical staining was conducted using an LSM 800 laser scanning microscope provided by Carl Zeiss (Oberkochen, Germany).

### 4.6. In Situ Hybridization (ISH)

Initially, RNA probes were developed to identify the localization and expression level of the selected genes. For their amplification, an original primer system was implemented:

- Zfp990 (ISH-Zfp990_fw 5′-GACCTCGAGTGGAGGAATGGGAATGTCTC-3′; ISH-Zfp990_rv-T3: 5′-AATTAACCCTCACTAAAGGGCGGCCGCAGATGGATCTGATGGGTAA GG-3′);
- Pcp4l1 (ISH-Pcp4l1_fw5′-GACCTCGAGGCGAGCTTAACACCAAAACA-3; ISH-Pcp4l1_rv-T3: 5′-AATTAACCCTCACTAAAGGGCGGCCGCGGAGCTGGAATCCTTTTTC C-3);
- Slc17a6 (ISH-Slc17a6_fw 5′-GACCTCGAGAAGAAGCAGGACAACCGAGA-3′; ISH-Slc17a6_rv-T3: 5′-AATTAACCCTCACTAAAGGGCGGCCGCGCAATGACTGCTCCAGCA TA-3′);
- Alpk1 (ISH-Alpk1_fw 5′-GACCTCGAGTGCTGGATCGTCTCTTGTTG-3′; ISH-Alpk1_rv-T3: 5′-AATTAACCCTCACTAAAGGGCGGCCGCCAGTATGCCCAGTGATGT GG-3′);
- Unc5d (ISH-Unc5d_fw 5′-GACCTCGAGTCCCGACTCTATCCCATCTG-3′; ISH-Unc5d_rv-T3: 5′-AATTAACCCTCACTAAAGGGCGGCCGCTCCATTCACGTAGACCACC A-3′).

Probe synthesis was conducted using T3 RNA polymerase (Roche, Basel, Switzerland) together with a DIG labeling mix (Roche, Switzerland). Later, probe purification was carried out using the RNase plus mini kit (Qiagen, Hilden, Germany) according to the manufacturer’s recommendations.

In situ hybridization was performed on frontal cryosections of the mouse brain (P21) with a thickness of 16 µm. Hybridization with RNA probes was carried out in a humid chamber with a solution of 50% formamide (NeoFroxx, Einhausen, Germany)/5× SSC (Sigma-Aldrich, Burlington, MA, USA) overnight at 65 °C. Subsequent washings were carried out three times in wash solution (50%formamide/1× SSC/0.1%/Tween-20 (PanReac AppliChem, Barcelona, Spain) at 65 °C and 1× MABT solution (100 mM Maleic acid (Sigma-Aldrich, Burlington, MA, USA), 150 mM NaCl (PanReac AppliChem, Spain), 0.1% Tween-20) and then blocked in a solution of 1× MABT with 10% goat serum (Gibco, Thermo Fisher Scientific, USA). Anti-DIG alkaline phosphatase antibody (1:1000 in blocking solution) (Roche, Switzerland) was then added and incubated at 4 °C overnight. It was then washed three times in 1×MABT solution and twice in pre-staining solution (0.1 M NaCl (PanReac AppliChem, Spain), 0.05 M MgCl2 (PanReac AppliChem, Spain), 0.1 M Tris base (PanReac AppliChem, Spain), pH 9.5, 0.05% Tween-20). NBT/BCIP (Roche, Switzerland) were added later into the staining solution (50% pre-staining solution/10% PVA (Sigma-Aldrich, Burlington, MA, USA). Staining was carried out in the dark for 3–16 h at 37 °C.

### 4.7. Fluorescent Ca^2+^ Measurements

The experiments were conducted during the daytime. Calcium measurements were performed using Fura-2/AM (Thermo Fisher Scientific, Waltham, MA, USA, Cat. #F1221), a ratiometric fluorescence calcium indicator, employing fluorescence microscopy. Neurons were loaded with the probe, which was dissolved in Hanks balanced salt solution (HBSS) consisting of the following concentrations: 156 mM NaCl, 3 mM KCl, 2 mM MgSO_4_, 1.25 mM KH_2_PO_4_, 2 mM CaCl_2_, 10 mM glucose, and mM 10 HEPES, pH 7.4. The final concentration of the probe was set at 5 μM, and the loading process was carried out at 37 °C for 40 min, followed by a 15 min washout period. The cells loaded with Fura-2 were then mounted on a coverslip and placed in the experimental chamber. To measure the concentration of free cytosolic Ca^2+^, the Carl Zeiss Cell Observer and an inverted motorized microscope Axiovert 200M were used in conjunction with a high-speed monochrome CCD-camera AxioCam HSm and a high-speed light filter replacement system, Ludl MAC5000. Fura-2 excitation and registration were recorded using a 21HE filter set (Carl Zeiss, Oberkochen, Germany) that included excitation filters BP340/30 and BP387/15, a beam splitter FT-409, and an emission filter BP510/90. The objective lens used was Plan-Neo fluar 10×/0.3, while the excitation light source was HBO 103W/2. Calcium responses were represented as the ratio of fluorescence intensities of Fura-2 excitation at 340 and 380 nm. Spontaneous Ca^2+^ activity was determined by the generation of Ca^2+^ signals by neurons during the recording time without any influence on the culture. Epileptiform Ca^2+^ activity was recorded in the form of Ca^2+^ signals that appeared after the application of a magnesium-free medium or bicuculline. When analyzing the images, the amplitude and period of Ca^2+^ pulses were determined for both spontaneous Ca^2+^ activity of neurons and induced epileptiform activity. Data analysis was performed using ImageJ 2002 software (RRID: SCR_003070).

To examine the development of the cell network, a Calcein AM fluorescent probe was introduced into the cell culture. This probe enables visualization of the cell bodies and their outgrowths. Calcein AM was loaded into HBSS solution and supplemented with 10 HEPES at a pH of 7.4 at a final concentration of 5 μM. Loading was performed at 37 °C for 40 min, followed by a 15 min washout period. To detect the fluorescence emitted by Calcein, an imaging system was utilized consisting of an inverted motorized microscope—Leica DMI6000B—and a high-speed monochrome CCD-camera—HAMAMATSU C9100. For the excitation and detection of Calcein fluorescence, the L5 filter cube (Leica Microsystems, Wetzlar, Germany) was employed. The cube incorporates excitation filters BP 480/40, a 505 dichromatic mirror, and an emission filter BP 527/30.

### 4.8. Extraction of RNA

The MagMAXmirVana Total RNA Isolation Kit (Thermo Fisher Scientific, Waltham, MA, USA, Cat. #A27828) was employed to extract the total RNA. To evaluate RNA quality, electrophoresis was conducted in the presence of 1 μg/mL ethidium bromide using a 2% agarose gel in Tris/Borate/EDTA buffer. The concentration of the extracted RNA was determined using a NanoDrop 1000c spectrophotometer. For reverse transcription of the total RNA, the RevertAid H Minus First Strand cDNA Synthesis Kit (Thermo Fisher Scientific, Waltham, MA, USA, Cat. #K1631) was utilized.

### 4.9. RNA Sequencing

RNA was isolated from “phenotype” and “without phenotype” *soc* mice brains using the Qiagen RNA easy mini kit (Qiagen, Germany). Following rRNA depletion, total RNA libraries were prepared using TruSeq stranded cDNA library (Illumina, San Diego, CA, USA) and sequenced on a Novaseq 6000 (Illumina, USA). Raw reads were aligned to GRCm38 using STAR [114] and quantified using TEcount [115] to count all non-coding and coding transcripts. Counts tables were then analyzed for differential expression using DESeq2 and volcano, and pseudo-Manhattan plots were generated in R.

### 4.10. Real-Time Polymerase Chain Reaction (RT-qPCR)

Each PCR was carried out using a 25 μL mixture composed of 5 μL of qPCRmix-HS SYBR (Evrogen, Moscow, Russia, Cat. #PK147L), 1 μL (0.2 μM) of the primer solution, 18 μL of RNase-free water, and 1 μL of cDNA. The DTlite Real-Time PCR System (DNA-technology, Moscow, Russia, 2017) was utilized for the amplification process. The amplification protocol consisted of an initial denaturation step at 95 °C for 5 min, followed by 40 cycles of denaturation at 95 °C for 30 s, annealing at 60–62 °C for 20 s, and extension at 72 °C for 20 s. A final extension step was performed at 72 °C for 10 min. The primer sequences were designed based on the analysis of nucleotide sequences of existing gene isoforms using FAST PCR 5.4 and NCBI Primer-BLAST software (https://www.ncbi.nlm.nih.gov/tools/primer-blast/primertool.cgi, accessed on 6 July 2023), ensuring specificity for the mouse. The obtained data were analyzed using DTlite software (https://dna-technology.com/sites/default/files/dtprime_dtlite_v06_part_2.pdf, accessed on 6 July 2023; DNA-technology, Moscow, Russia) and Origin 8.5 software (OriginLab Corporation, Northampton, MA, USA). The expression of the genes under investigation was normalized to the gene encoding Glyceraldehyde 3-phosphate dehydrogenase (GAPDH) and compared to “without phenotype” *soc* mice. The data were analyzed using Livak’s method [116].

### 4.11. Statistical Analysis

All values are presented as mean ± standard error (SEM), mean ± standard deviation (SD), or as individual Ca^2+^-signals. The data presented in this study were obtained from a minimum of three separate cell cultures, each from two to three different passages. Statistical analyses were conducted using paired *t*-test for data that distributed normally and the Mann–Whitney test to compare two groups of data without normal distribution. MS Excel (Microsoft Office 2016, Redmond, Washington, DC, USA) and ImageJ (available at https://imagej.nih.gov/ij/download.html, accessed on 18 May 2023), which utilized Java 1.6.0_12 (RRID: SCR_003070) developed by LOCI at the University of Wisconsin, Madison, WI, USA, were used. Additionally, Origin 2016 (OriginLab, Northampton, MA, USA) and Prism GraphPad 7 (GraphPad Software, RRID: SCR_002798) were employed for data analysis and statistical calculations.

## 5. Conclusions

Using ENU-directed mutagenesis, it was possible to obtain a new strain of mice with signs of epileptogenesis and identify the locus in which the mutation appeared and became established. To characterize this mouse strain, a comprehensive approach was used to analyze the epileptiform activity of cultured neurons and the expression of genes encoding proteins involved in the genesis of epilepsy. It turned out that both in vitro and in vivo, the soc mouse strain is characterized by impaired expression of excitatory NMDA receptors, phosphoinositol 3-kinase, and membrane ion channels.

## Data Availability

The data presented in this study are available upon request from the corresponding author.

## Supplementary Materials

The following supporting information can be downloaded at: https://www.mdpi.com/article/10.3390/ijms242317104/s1.
