# Supplementary material for: Socrates: A Novel N-Ethyl-N-nitrosourea-Induced Mouse Mutant with Audiogenic Epilepsy
Source: Int J Mol Sci. 2023 Dec 4;24(23):17104. doi: 10.3390/ijms242317104 (PMC10707124; doi:10.3390/ijms242317104)
Supplement: Supplementary file 1 [file ijms-24-17104-s001.zip › ijms-2709971-supplementary.pdf]

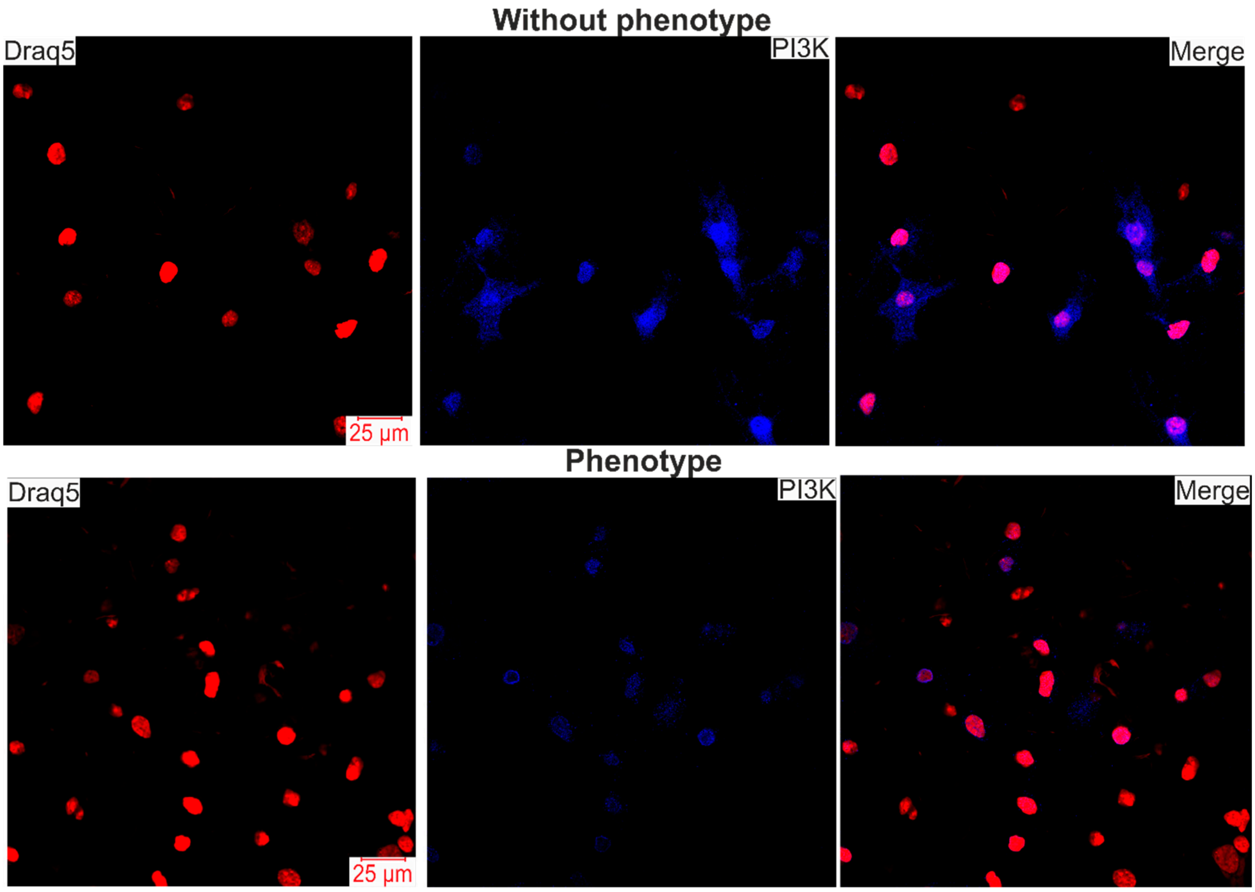

Figure S1. Immunocytochemical staining of cortical neurons derived from without phenotype and phenotype mice with antibodies against phosphoinositide 3-kinase (PI3K). Nuclei staining with Draq5.

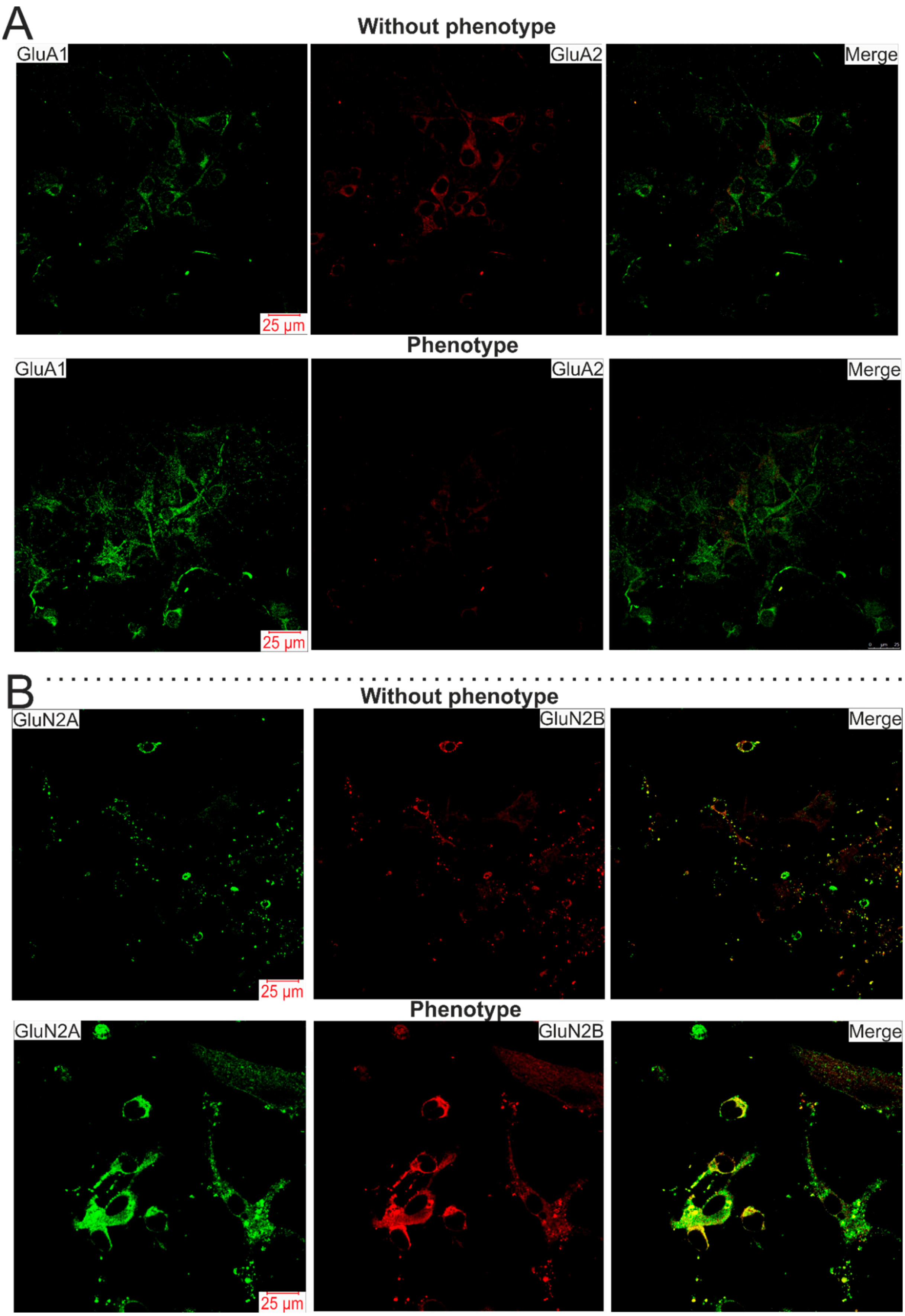

Figure S2. Immunocytochemical staining of cortical neurons derived from without phenotype and phenotype mice with antibodies against GluA1- and GluA2-subunits of AMPAR (A) and against GluN2A and GluN2B-subunits of NMDAR (B). Nuclei staining with Draq5.
